# Interpretable ensemble remaining useful life prediction enables dynamic maintenance scheduling for aircraft engines

**DOI:** 10.1038/s41598-025-23473-2

**Published:** 2025-11-13

**Authors:** Hikmetcan Özcan

**Affiliations:** Department of Computer Engineering, Kocaeli University, Umuttepe Campus, Kocaeli, 41380 Kocaeli Türkiye

**Keywords:** Aircraft Engines, Dynamic Maintenance Scheduling, Hybrid Ensemble Models, Predictive Maintenance, Remaining Useful Life Prediction, Engineering, Mathematics and computing

## Abstract

This study introduces a predictive-maintenance framework for aircraft engines that integrates accurate remaining-useful-life (RUL) estimation with a cost-aware scheduling strategy. The predictive layer employs ensemble learning by combining LightGBM, CatBoost, and Gradient Boosting, thereby enhancing both accuracy and stability. The scheduling layer initiates maintenance once the predicted RUL falls below a tunable threshold $\tau$ (set to 15 cycles in experiments) and allocates service slots under cost and risk tolerance constraints, ensuring flexibility for conservative operation when necessary. The framework is evaluated on the NASA C-MAPSS dataset (FD001–FD004), covering single- and multi-condition as well as single- and multi-fault scenarios. Experimental results demonstrate strong performance on FD001 and FD003, with competitive results on FD002 and FD004. For instance, the LightGBM+CatBoost ensemble achieves RMSE = 6.62 and RUL Score = $2.951 \times 10^{3}$ on FD001, while the three-model ensemble yields RMSE = 9.71 and RUL Score = $1.037 \times 10^{4}$ on FD003. To ensure transparency and reliability, SHAP-based interpretability analysis is applied, highlighting critical sensors and operational cycles. The ensemble approach provides more balanced attributions, which enhances auditability and supports engineering validation in safety-critical domains. Overall, this study contributes to aviation predictive maintenance by delivering robust and interpretable RUL predictions and by translating them into tunable maintenance policies; however, these contributions are demonstrated on C-MAPSS and with a fixed illustrative threshold, so the reported gains should be interpreted as benchmark-specific until confirmed on real fleets and under alternative risk-aware policies.

## Introduction

The aviation industry is a critical sector where technical maintenance processes must be continuously improved due to the requirements for high reliability, efficiency, and cost optimization. In this sector, aircraft engines, in particular, stand out as one of the most essential components in terms of overall system performance and reliability. Engine failures not only lead to operational disruptions and high maintenance costs but also pose significant risks to flight safety. In this context, the effective management of maintenance processes for aircraft engines is indispensable, not only for optimizing operational costs but also for ensuring operational safety and continuity^1,2^.

Traditional maintenance methods typically adopt a time-based approach, scheduling maintenance activities according to predetermined fixed intervals or flight hours. However, since these methods do not offer the capability to assess maintenance needs based on real-time data, they may result in both unnecessary maintenance actions and the inability to predict potential critical failures^3^. These limitations highlight the need for more dynamic and intelligent management of maintenance processes, especially in safety-critical and cost-sensitive sectors like aviation.

PdM is an innovative approach that has emerged in response to these requirements and has been extensively researched in recent years. PdM predicts the RUL of system components and analyzes maintenance needs in real time by utilizing ML algorithms and data analytics techniques. This approach not only enables the prediction of failures but also optimizes maintenance operations, thereby enhancing operational efficiency and reducing costs^4,5^.

In this study, an innovative PdM framework is proposed that utilizes ML ensemble methods for RUL prediction and dynamic maintenance scheduling in aircraft engines. Ensemble models combine the strengths of different ML algorithms to enhance prediction accuracy and provide more reliable results. In addition to algorithms such as LightGBM, CatBoost, Gradient Boosting, and XGBoost, methods like SVM, KNN, and LR are also employed and integrated using ensemble learning techniques. Furthermore, an advanced dynamic maintenance scheduling algorithm analyzes the predicted RUL values, enabling maintenance activities to be planned in a more flexible and optimized manner. This innovative framework aims to significantly improve aircraft engine maintenance processes in terms of both cost efficiency and operational reliability.

The remainder of this article is organized as follows. Section 2 states the problem, motivates the study, and summarizes the original contributions. Section 3 surveys related work on predictive maintenance and dynamic maintenance scheduling. Section 4 details the C-MAPSS dataset, preprocessing, the hybrid ensemble models, and the dynamic maintenance scheduling algorithm. Section 5 presents the experimental results together with ablation and interpretability analyses, and discusses comparisons with baselines. Section 6 concludes with key findings, limitations, and directions for future work.

## Motivation and contribution

In aircraft engine maintenance management, reducing operational costs and enhancing flight safety are among the critical objectives. However, since most existing maintenance processes rely on static and time-based approaches, they not only generate unnecessary costs but also fall short in preventing potential failures^2^. In this context, PdM methods promise to overcome these shortcomings by optimizing maintenance operations using real-time, data-driven decision-making mechanisms. Nevertheless, many existing PdM applications employ prediction methods based on a single ML model, which have limitations in terms of accuracy and reliability^6^.

A detailed review of recent studies on ensemble approaches for predictive maintenance and dynamic maintenance scheduling is provided in Table 1, highlighting the main techniques, datasets, and performance outcomes. This analysis reveals specific gaps — such as limited model diversity, lack of dynamic scheduling integration, and insufficient validation on complex datasets — which directly motivate the present work.

This study presents the following four main motivations and contributions to improve PdM processes:

1. **High Prediction Performance with Hybrid Ensemble Models:** Ensemble learning techniques, which combine the strengths of different ML algorithms, offer an effective approach to overcome the limitations of individual models and improve prediction accuracy. In this study, hybrid ensemble models were developed by integrating powerful models such as LightGBM, CatBoost, Gradient Boosting, and XGBoost, along with other methods like SVM and Linear Regression. Across the C -MAPSS **FD001–FD004** subsets, the proposed ensembles consistently outperform single models; in particular, they reach strong results on **FD001** and **FD003** (e.g., FD001: $R^2{=}0.9904$, $\textrm{MSE}{=}43.79$; FD003: $R^2{=}0.9904$, $\textrm{MSE}{=}94.21$), while remaining competitive on FD002 and FD004. These findings indicate the potential of ensemble models to provide high accuracy and consistency in PdM processes^5^.
2. **Optimization of Maintenance Processes with a Dynamic Scheduling Algorithm:** Unlike traditional time-based maintenance approaches, this study proposes a dynamic maintenance scheduling algorithm that analyzes predicted RUL values and plans maintenance needs in real time. The algorithm employs a *threshold-based* policy that triggers maintenance when $\widehat{\textrm{RUL}} \le \tau$ (illustratively $\tau {=}15$ cycles), and can optionally incorporate a *risk tolerance* parameter $\alpha$ for quantile-based triggering. In practice, it selects cost-aware slots to minimize expected operational impact, thereby preventing unnecessary interruptions and improving efficiency^4,7^.
3. **A Practical and Realistic PdM Framework:** The C -MAPSS dataset used in this study provides simulation data reflecting real operational conditions of aircraft engines. The proposed hybrid models and the dynamic scheduling algorithm are *validated on all four sub-datasets (FD001–FD004)*, supporting applicability across varying operating conditions and fault modes. The developed method also has features that allow integration into aviation maintenance strategies^7,8^.
4. **Improving Stability and Interpretability Through Hybrid Integration:** The framework combines ensemble models with a dynamic maintenance algorithm to achieve robust and stable predictions. In addition, *SHAP-based* global and local analyses reveal more balanced feature attributions in the ensembles than in single models, strengthening model transparency and practitioner trust in safety-critical decision-making.

These contributions, as summarized relative to prior studies in Table 1, demonstrate how the proposed framework addresses the identified research gaps and advances the state of the art in predictive maintenance for aviation.

In conclusion, this study presents an innovative approach to PdM processes in the aviation industry, providing significant benefits in terms of both cost efficiency and operational reliability. Future studies may aim to apply these methods to larger datasets and integrate them with deep learning techniques for implementation in larger-scale operational systems. This would enable more effective management of predictive maintenance processes for aircraft engines and other critical systems^5–7,9^.

## Literature review

### Fundamentals and advantages of predictive maintenance

Jardine and his team conducted a comprehensive review of PdM applications. The study thoroughly examines the fundamental building blocks of PdM systems, the algorithms used, and the advantages these methods offer, particularly in high-risk sectors like aviation. The research emphasizes that PdM can reduce costs and enhance operational reliability by enabling failure prediction and optimizing maintenance processes. Notably, the study highlights the positive impact of PdM on system reliability by transforming maintenance processes into a proactive approach. However, while the research addresses the effectiveness of PdM in failure prediction and maintenance optimization, it does not cover the contribution of dynamic scheduling algorithms to operational processes^10^.

In his study detailing the fundamentals of PdM, Mobley addresses the advantages of PdM compared to traditional time-based maintenance methods. The study illustrates how PdM optimizes costs and enhances operational reliability by improving the effectiveness of failure detection and prevention processes. Mobley also explains the key sensor technologies and analytical methods used to integrate PdM into the maintenance cycle. However, his research does not focus on the application of hybrid ensemble models aimed at improving the accuracy of RUL prediction in aircraft engines^11^.

Tsang systematically analyzes the key advantages of PdM, focusing on cost optimization, failure prevention, and business continuity. He details the positive impact of PdM on workplace safety and system performance compared to traditional maintenance methods. The study also emphasizes how PdM drives transformation in high-risk industries. However, his research does not address the performance evaluation of hybrid ensemble models for RUL prediction in aircraft engines or the impact of dynamic maintenance scheduling algorithms on operational disruptions^12^.

### RUL prediction and data-driven algorithms

Zhu and colleagues have comprehensively examined the applicability of deep learning algorithms in the intelligent fault diagnosis of rotating machinery. Their study demonstrates that deep learning models offer higher prediction accuracy compared to traditional machine learning methods and provide significant advantages in predictive maintenance processes, particularly through automatic feature extraction. However, this research does not delve into the performance improvements offered by hybrid ensemble models compared to individual algorithms, nor does it evaluate the impact of dynamic maintenance planning algorithms on operational costs. Additionally, it does not provide an in-depth analysis of the integration of machine learning algorithms into complex systems such as aircraft engines^13^.

Pérez-Ruiz and his team have developed an integrated monitoring, diagnosis, and prognostics system for aircraft engines subjected to long-term performance degradation. This study offers a comprehensive approach that involves the interplay of feature extraction, anomaly detection, fault identification, and prognostics algorithms. Notably, the system has been shown to maintain fault detection, identification, and prediction accuracy even in the face of long-term engine degradation. However, the research does not provide an in-depth analysis of the integration of hybrid models or the effectiveness of ensemble methods^14^.

Pang and colleagues proposed a method based on incremental capacity analysis (ICA) and Gaussian process regression (GPR) to estimate the RUL of lithium-ion batteries. This study involves extracting health indicators from charge and discharge cycles to monitor battery health and predict future features using support vector regression. Additionally, a Gaussian process regression model was employed alongside Shapley Additive Explanation (SHAP) analysis to perform capacity predictions. Notably, the proposed method effectively captured capacity regeneration and provided accurate RUL predictions^15^.

### C-MAPSS dataset and PdM applications

Saxena and his team introduced the C-MAPSS dataset, which is widely used to model PdM processes, and highlighted its reliability as a resource for testing and developing RUL prediction models in aircraft engines. The study emphasized the critical role of the C-MAPSS dataset in enabling more accurate predictions and optimized maintenance processes in PdM applications. However, their research did not focus on the performance analysis of hybrid ensemble models or the integration of dynamic maintenance scheduling algorithms using the C-MAPSS dataset. Additionally, the impact of combining different machine learning methods on RUL prediction accuracy was not evaluated^16^.

Saxena and Goebel developed prediction models using the C-MAPSS dataset that reflect the complex structure and real operational conditions of aircraft engines. Their study provided a reliable foundation for RUL prediction and offered significant insights to enhance the applicability of PdM in complex systems like aircraft engines. However, their research did not address the performance improvements achieved through hybrid ensemble models or evaluate the impact of integrating dynamic maintenance scheduling algorithms on operational processes. Additionally, no analysis was provided on how prediction accuracy could be improved by combining different machine learning techniques instead of relying on individual models^17^.

Zhang and colleagues have developed a method to address the issue of data scarcity in estimating the RUL of industrial equipment. Specifically, when working with limited datasets such as the C-MAPSS dataset, the proposed Convolutional Recurrent Generative Adversarial Network (CR-GAN) model aims to enhance existing RUL prediction methods by generating realistic time series data. This approach improves prediction accuracy through data augmentation by capturing both non-cyclic and cyclic degradation patterns. However, the study does not delve into the integration of hybrid ensemble models or the applicability of dynamic maintenance planning algorithms^18^.

Faizanbasha and Rizwan proposed a deep learning–stochastic ensemble framework for RUL prediction and predictive maintenance, integrating dynamic mission abort policies to improve decision-making in safety-critical operations. The proposed approach was evaluated on the NASA C-MAPSS dataset, where it demonstrated competitive accuracy and robustness compared to conventional deep learning approaches. These results highlight the potential of combining stochastic ensemble learning with operational policy optimization in PdM applications. However, the study does not present a comprehensive hybrid ensemble structure integrating diverse machine learning algorithms, nor does it include a dedicated dynamic maintenance scheduling algorithm with threshold-based decision-making and cost–risk analysis as proposed in this work^19^.

Qin and his team developed a spatio-temporal multi-sensor information fusion network with prior knowledge embedding, designed to enhance RUL prediction. The approach integrates spatial correlations among sensors with temporal degradation patterns, while incorporating domain knowledge to improve predictive performance. The method was validated on the NASA C-MAPSS, and SCADA datasets, achieving superior accuracy compared to several baseline models. However, the study, while leveraging dynamic temporal-based graph construction, does not incorporate a hybrid ensemble strategy that could integrate its dynamic graph learning with other complementary machine learning or deep learning models^20^.

### The role of hybrid models in PdM applications

Hu and his team developed hybrid models by combining different data-driven prognostic algorithms and examined their effectiveness in PdM processes. The study demonstrated that hybrid models provide higher accuracy and robustness compared to a single algorithm, thereby enhancing the overall performance of PdM applications. However, the research did not offer an evaluation of the integration of dynamic maintenance scheduling algorithms with hybrid models or assess the impact of this integration on operational costs and disruptions. Furthermore, a detailed analysis of the applicability of hybrid models in complex systems such as aircraft engines was not included^5^.

Zhang and colleagues have comprehensively examined the applicability of deep learning methods for fault diagnosis in rotating machinery. Their study demonstrates that deep learning models, particularly in automatic feature extraction and fault classification from vibration signals, outperform traditional methods. In particular, models such as CNN and Recurrent Neural Networks were highlighted as effective for fault diagnosis in rotating machinery. However, the research does not provide a detailed analysis of the performance of hybrid deep learning models compared to traditional machine learning methods or the effectiveness of ensemble models in estimating the RUL of aircraft engines^21^.

Wang and his team developed a new methodology to enhance the performance of hybrid models in PdM applications, aiming to improve the robustness of RUL prediction by combining both ML and statistical approaches. However, the integration of the developed methodology with dynamic maintenance scheduling algorithms and the impact of this integration on operational processes were not addressed in the study^22^.

Alomari and colleagues developed an interpretable hybrid approach for predicting the RUL of aircraft engines. Their study integrated principal component analysis (PCA)-based dimensionality reduction with ensemble models such as XGBoost, Random Forest, and Multi-Layer Perceptron (MLP), achieving high prediction accuracy. Additionally, they calculated aggregated feature importance scores to enhance model interpretability and transparency in predictive maintenance systems. However, their work does not provide a detailed comparative analysis of different ensemble structures, nor does it explore the integration of predictive models with dynamic maintenance scheduling algorithms. In this regard, this study offers a novel contribution by combining hybrid ensemble learning with a real-time dynamic maintenance planning algorithm and conducting comprehensive statistical comparisons between various ensemble configurations^23^.

### Dynamic maintenance scheduling algorithms

Zhou and his colleagues investigated the impact of dynamic scheduling algorithms in PdM processes and developed an algorithm to optimize maintenance scheduling based on the remaining useful life of machine components. The study revealed that dynamic scheduling algorithms can enhance system efficiency while reducing unnecessary maintenance costs. This approach aims to minimize operational disruptions by offering a more flexible and adaptive strategy for PdM processes. However, the study did not provide any recommendations regarding the use of ensemble learning methods to improve prediction accuracy^8^.

Khan and Yairi examined methods for optimizing maintenance needs by integrating dynamic scheduling algorithms into PdM processes and detailed the algorithm’s ability to work with real-time data and its impact on reducing maintenance costs. However, the integration of dynamic scheduling algorithms with hybrid ensemble models and the potential applications of this integration in complex systems such as aircraft engines were not explored^24^.

Zonta and colleagues conducted a systematic literature review to examine PdM applications in the context of Industry 4.0. Their study highlights the critical role of predictive maintenance in enhancing flexibility and operational efficiency in manufacturing systems. Specifically, they emphasized the importance of dynamic planning algorithms in optimizing maintenance operations in real-time systems. However, the research does not provide a detailed analysis of the integration of dynamic planning algorithms with hybrid ensemble models or the applicability of this approach to complex systems such as aircraft engines^25^.

### Summary of existing works

Table 1 provides a comprehensive comparison of key studies in the fields of PdM and Dynamic Maintenance Scheduling. The table summarizes the methodologies employed, the key findings, and how these studies contribute to the ongoing advancements in these areas. PdM approaches focus on improving the accuracy and reliability of RUL predictions by utilizing various methods, including ML algorithms, DL techniques, TS analysis, and MLP. On the other hand, dynamic maintenance scheduling algorithms aim to optimize maintenance tasks, enhancing system efficiencies and reducing unnecessary costs. In this context, studies in both areas strive to significantly improve the effectiveness of maintenance processes.

**Table 1:** Overview of key predictive maintenance and dynamic maintenance scheduling works, summarizing methodologies, findings, and the novel contributions of this study.

Ref.	Application Domain	Methodology	Key Findings
^5^	PdM	MLP-based RUL prediction	Improved RUL prediction accuracy with optimized parameters
^8^	Dynamic scheduling	Dynamic scheduling algorithms for maintenance optimization based on RUL	Enhanced system efficiency and reduced unnecessary maintenance costs
^13^	PdM	Deep learning based algorithms (DBN, AE, CNN, RNN, GAN)	High accuracy in fault diagnosis, challenges with small datasets
^14^	PdM	Integrated PHM system (feature extraction, anomaly detection, fault diagnosis, prognostics)	Resilient to long-term performance deterioration, low computational cost
^15^	PdM	ICA + GPR	High sensitivity to degradation, challenges with unmeasurable health indicators
^16^	PdM	Damage propagation modeling using C-MAPSS simulator	Simulated degradation and flow losses in aircraft engines over time
^18^	PdM	CR-GAN-based time-series generation for RUL estimation	Enhanced RUL estimation by augmenting datasets with realistic synthetic data
^19^	PdM	Deep learning–stochastic ensemble integrated with dynamic mission abort policies	Competitive accuracy and robustness; effective in safety-critical PdM
^20^	PdM	Spatial–temporal multi-sensor fusion network with prior knowledge embedding, evaluated on C-MAPSS and PRONOSTIA	Improved RUL prediction accuracy by capturing temporal dependencies and spatial correlations among sensors
^21^	PdM	Deep learning-based fault diagnosis methods (DBN, AE, CNN, RNN, GAN)	Comprehensive review of deep learning techniques for fault diagnosis in rotating machinery
^22^	PdM	Hybrid prognostics approach for RUL estimation of rolling element bearings	Combines multiple prognostic models to improve RUL prediction accuracy
^23^	PdM	Feature engineering + aggregated feature importance	Enhanced RUL prediction interpretability for aircraft engines
^24^	Dynamic scheduling	Deep learning-based SHM applications	Review of DL applications in SHM, highlighting benefits and limitations.
^25^	PdM+ Dynamic scheduling	Systematic literature review and PdM taxonomy	Classification of PdM methods in the context of Industry 4.0 and discussion of current challenges
**This Study**	PdM + Dynamic Scheduling (C-MAPSS)	Hybrid ensemble; threshold-based policy (Alg. 1; $\tau$/optional $\alpha$; cost-aware selection)	Competitive on FD001-FD004; $\tau /\alpha$-tunable safety–cost policy (quantile triggering, cost-aware);

### Research gaps in the literature and the innovative contributions of this study

Upon reviewing the literature, it is observed that most studies focus on single machine learning models, which are limited in terms of generalization capability and prediction accuracy. Specifically, research on the integration of ensemble learning models into predictive maintenance applications remains limited. In this study, hybrid ensemble models combining powerful algorithms such as LightGBM, CatBoost, Gradient Boosting, and XGBoost have been utilized to achieve more balanced and reliable predictions. Additionally, a dynamic maintenance scheduling algorithm that continuously analyzes the predicted RUL values has been developed and thoroughly tested using the FD001 subset of NASA’s C-MAPSS dataset. Thus, this study addresses the gaps in the literature and provides an innovative solution in the field of predictive maintenance by enhancing both prediction accuracy and operational reliability.

## Materials and methods

This section details the methods and data sources used to optimize the PdM processes of aircraft engines. The C-MAPSS dataset used in the study enables the development and performance evaluation of ML models. The hybrid model and dynamic maintenance scheduling algorithms developed in this study aim to enhance the accuracy and consistency of PdM processes. In high-dimensional datasets, optimized feature selection significantly affects the training performance of ML models. Recent studies have shown that strategic feature selection significantly improves both accuracy and computational efficiency in complex systems^26^. In this context, the methodological framework of the study is presented by detailing the dataset characteristics, model development process, and evaluation metrics.

### Development environment

The development, implementation, and analysis of the ML algorithms used in this study were carried out on a computer with the following system and software specifications:

- **Operating System**: Windows 10 Enterprise LTSC 64-bit (10.0, Build 19044)
- **Processor**: Intel(R) Xeon(R) Platinum 8358P CPU @ 2.60GHz (6 Cores)
- **RAM**: 24,576 MB
- **Programming Language**: Python 3.12.4
- **Development Environment**: Jupyter Notebook

### C-MAPSS dataset

In this study, the C-MAPSS (Commercial Modular Aero-Propulsion System Simulation) dataset, provided by NASA Ames Research Center, was used to support PdM applications for aircraft engines^17^. The C-MAPSS dataset offers simulated data in four different modes to monitor the performance and failure tendencies of aircraft engines under various operating conditions. This dataset is widely used for the development of PdM models and the prediction of the RUL of aircraft engine components.

The C-MAPSS dataset consists of four sub-datasets (FD001–FD004) representing the performance and operational scenarios of different engines of the same turbofan engine type. It contains 21 different sensor measurements that enable the analysis of aircraft engine wear and degradation processes. These sub-datasets are designed to simulate different engine groups and flight conditions. As summarized in Table 2, FD001 represents a simple scenario with constant operating conditions and a single fault mode, while FD002 includes multiple operating conditions with a single fault mode. FD003 is structurally similar to FD001 but simulates multiple fault modes. FD004 presents the most complex scenario, combining multiple operating conditions with multiple fault modes^20,27^. In this study, all sub-datasets were analyzed to comprehensively evaluate the proposed method’s performance across scenarios of varying complexity levels.

**Table 2:** Summary of C-MAPSS sub-datasets FD001–FD004^20^.

Dataset	Number of Engines (Train/Test)	Operating Conditions	Fault Modes
FD001	100/100	1	1
FD002	260/259	6	1
FD003	100/100	1	2
FD004	248/249	6	2

The hybrid model and dynamic maintenance scheduling algorithm used in this study were developed based on the sensor data and operating condition data obtained from the C-MAPSS dataset.

### Data preprocessing

Before applying the C-MAPSS dataset to ML models, several preprocessing steps were conducted to improve data quality and enhance prediction performance. These steps were selected based on well-established practices in the PdM literature, with the objective of ensuring that the dataset was both reliable and optimally structured for the subsequent modeling phase.

In this study, all four sub-datasets of the NASA C-MAPSS dataset (FD001, FD002, FD003, and FD004) were utilized to evaluate the proposed approach under different operating conditions and fault modes. Each sub-dataset contains multi-sensor time series data for multiple engines, recorded from the start of operation until failure, resulting in sequences of varying lengths. All available 21 sensor measurements and operating condition variables were used as model inputs without removing any sensors. The training and test sets were assigned according to the official train–test engine allocation provided with the C-MAPSS dataset, as is common in the literature, to ensure comparability with previous studies.

The first step involved handling missing or faulty data, which can significantly reduce the accuracy of PdM models. Missing values were addressed using common imputation strategies, such as replacing them with the mean or median of the corresponding feature, or removing incomplete entries when necessary. These methods are widely recognized for their ability to preserve data integrity and maintain predictive accuracy across a variety of industrial datasets^28^.

Following data cleaning, scaling and normalization procedures were applied to harmonize the range and distribution of sensor readings and operating condition variables. Techniques such as min–max scaling and z-score standardization were implemented to ensure that all features were on comparable scales. This preprocessing stage was particularly important for algorithms sensitive to feature magnitude, including logistic regression, SVM, and KNN, where consistent scaling contributes to improved model stability and accuracy^29^.

In terms of sequence length differences among engines, each operational cycle was labeled with its corresponding RUL value. This cycle-based labeling inherently manages engines with short or long operational histories without requiring truncation or padding of the time series. Thus, the model learns from all available operational data while preserving the chronological structure of each engine’s life.

Collectively, these preprocessing steps provided a robust foundation for model development. By addressing data quality issues, ensuring consistent feature scaling, and organizing the full set of sensor and operating condition variables in a cycle-based RUL-labeled format, the dataset was effectively prepared for accurate and reliable failure prediction in PdM applications.

### Machine learning process and dynamic maintenance algorithm

**Fig. 1:**
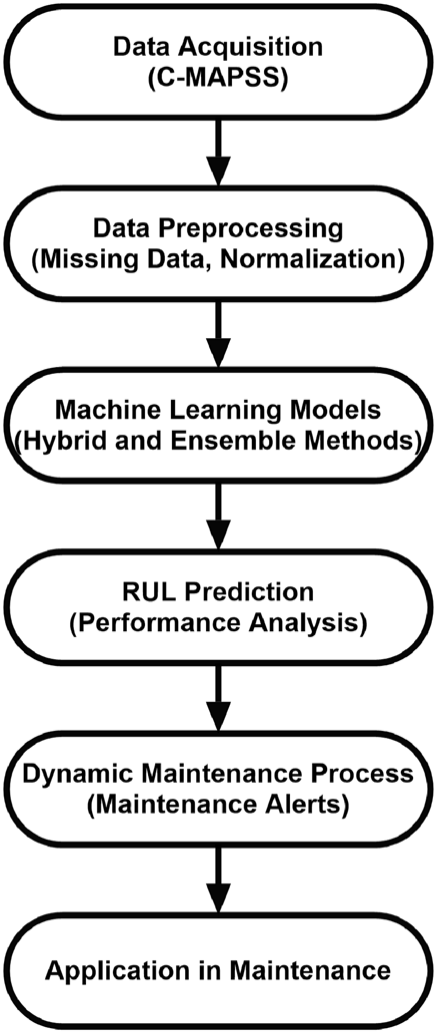
End-to-end workflow of the proposed predictive maintenance approach, including data acquisition, preprocessing, hybrid model training, RUL prediction, and dynamic scheduling decisions.

The overall process of the methods used in this study is illustrated in Figure 1. The process begins with data acquisition, followed by data preprocessing, and then focuses on training machine learning models. The RUL predictions obtained from the models are integrated with the dynamic maintenance algorithm to determine maintenance requirements. Finally, the results obtained from these maintenance processes are analyzed, and recommendations are developed.

### Determination of dynamic maintenance scheduling threshold

In this study, the maintenance trigger in the Dynamic Maintenance Scheduling (DMS) algorithm is instantiated as $\tau =15$ cycles for reporting and illustration; however, $\tau$ is not a universal constant but a policy variable that can be specified by the operator or OEM and adapted to engine family, operating regime, and asymmetric maintenance costs.

The rationale for this choice rests on three pillars: (i) reliability theory and Weibull-type life models show that the hazard rate increases markedly at low RUL, supporting the rationality of preventive maintenance at a low-RUL threshold^30,31^; (ii) historical evidence such as the C-MAPSS dataset indicates that failures tend to cluster within specific late-life cycle ranges^17^; and (iii) from a safety–cost perspective, performing planned maintenance as RUL approaches critical levels preserves flight safety and operational continuity while optimizing total cost^32^. To ground this framing empirically, Table 17 reports illustrative alert outcomes produced by different models under $\tau =15$.

This study’s decision rule—“trigger maintenance when $\widehat{RUL}\le \tau$”—is compatible with both static and adaptive policies. In practice, $\tau$ may be derived from multiple information sources without altering the predictive layer, including OEM-prescribed inspection/overhaul cycles, safety-margin indicators (e.g., vibration or EGT margin exceedances), fleet readiness and mission-window constraints, late–early maintenance cost ratios, operating conditions (altitude/temperature/load profiles), and predictive uncertainty (e.g., quantile-based triggering using the distribution of $\widehat{RUL}$).

*Safety–cost implications of alert errors (FP/FN).* Predictive-maintenance alerts entail asymmetric operational and safety consequences. False positives (FPs) may trigger early or unnecessary maintenance, leading to no-fault-found removals, avoidable parts/labor usage, and additional ground time; false negatives (FNs) defer maintenance beyond the safe window, increasing the probability of safety-margin excursions (e.g., vibration/EGT exceedances) and operational disruptions (AOG, unscheduled removal). Accordingly, this study treats the maintenance trigger $\tau$ and the risk tolerance $\alpha$ (Algorithm 1) as tunable policy variables that shape the FP/FN trade-off under operator-specific cost ratios and regulatory constraints. Concretely, the decision rule operates on $\hat{\textrm{RUL}}$ and supports quantile-based triggering to enforce $P_{\textrm{breach}}\!\le \!\alpha$, together with cost-aware slot selection that minimizes the expected operational objective. Table 17 illustrates the expected directionality: increasing $\tau$ (or decreasing $\alpha$) typically reduces predicted FNs at the expense of more FPs; tighter alerting (lower $\tau$ or higher $\alpha$) has the opposite effect. This framing links the safety objective (avoiding FNs) with the economic objective (limiting FPs) and makes the policy explicit and auditable^33–37^.

Operationally, the threshold policy in this subsection is implemented end-to-end by Algorithm 1; Table 17 shows an example instantiation of that algorithm with two base models (LightGBM, CatBoost) and $\tau _{\text {maint}}{=}15$ cycles.

**Algorithm 1:**
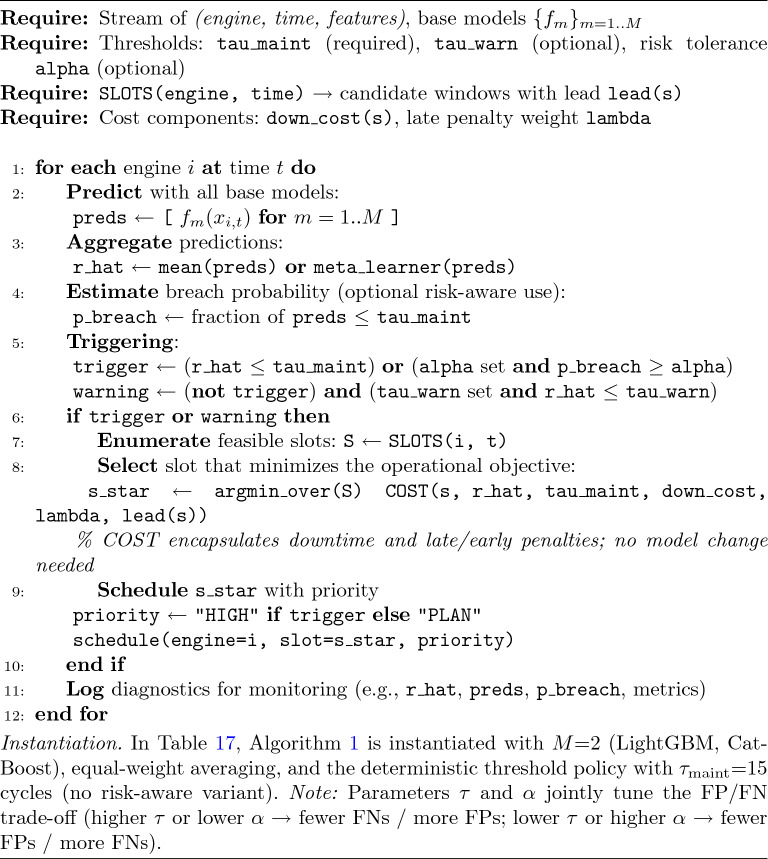
Dynamic Maintenance Scheduling with Ensemble RUL

### Hybrid modeling and ensemble learning methods

Hybrid modeling and ensemble methods were designed to combine the strengths of machine learning algorithms and improve model accuracy. The details of the models used in the study are presented in Table 3.

**Table 3:** Overview of machine learning algorithms applied in the hybrid ensemble modeling process, highlighting their core functions and characteristics.

Model name	Short description	References
XGBoost	An accelerated and optimized version of the gradient boosting method.	^38^
Gradient Boosting Regressor (GBR)	A gradient boosting algorithm that reduces the errors of weak learners.	^39^
SVM	Separates data with maximum-margin hyperplanes; via kernels (e.g., RBF) it captures non-linear structure.	^40^
Linear Regression	Fits a linear mapping via ordinary least squares (OLS); a strong baseline for continuous RUL prediction.	^41^
CatBoost	A gradient boosting algorithm that stands out with its categorical data processing capabilities.	^42^
LightGBM	A fast and efficient gradient boosting model with a lightweight structure and leaf-based tree growth.	^43^
KNN	A non-parametric method that uses the features of the nearest neighbors for prediction.	^44^
MLP	A multi-layer artificial neural network that models non-linear relationships.	^45^
Bayesian Ridge and Ridge Regression	Ridge reduce overfitting with the L2 norm; Bayesian Ridge can model parametric uncertainties.	^46^

**Table 4:** Hyperparameter configurations selected via Grid Search CV for each machine learning model used in the study.

Model name	Applied parameters (Grid search CV)
XGBoost	objective=’reg:squarederror’, learning_rate=0.2, max_depth=4, n_estimators=200, subsample=1.0, colsample_bytree=0.8, random_state=42
Gradient Boosting Regressor	n_estimators=300, max_depth=7, learning_rate=0.1, subsample=0.8, random_state=42
Support Vector Machine (SVM)	C=500, epsilon=0.5, gamma=’scale’, kernel=’rbf’
Linear Regression (LR)	Features(degree=1), LinearRegression(fit_intercept=True)
LightGBM (LGBM)	n_estimators=500, learning_rate=0.1, max_depth=−1, num_leaves=31, subsample=0.8, colsample_bytree=0.8, random_state=42
CatBoost (Cat)	iterations=500, learning_rate=0.1, depth=6, random_seed=42, verbose=0
MLP	hidden_layer_sizes=(50, 50, 50), activation=’relu’, solver=’adam’, alpha=0.01, learning_rate=’constant’, max_iter=500, random_state=42
K-Nearest Neighbors	n_neighbors=14, weights=’uniform’, metric=’manhattan’
Ridge Regression	alpha=1, max_iter=1000, solver=’auto’, random_state=42
Bayesian Ridge Regression	alpha_1=1e-6, alpha_2=1e-5, lambda_1=0.0001, lambda_2=1e-6, tol=0.0001
Random Forest	n_estimators=100, max_depth=14, min_samples_split=2, min_samples_leaf=1, random_state=42, n_jobs=−1

### Parameters used in models and optimization process

The hyperparameters of the machine learning models used in this study were determined using the Grid Search CV method to optimize model performance. Grid Search CV systematically tests different combinations of hyperparameters and employs a cross-validation approach to select the parameters that provide the best performance. The final parameters applied for each model are listed in Table 4.

### Advantages and disadvantages of ensemble models

Ensemble models are widely applied in machine learning to enhance predictive accuracy and improve generalization capacity. By combining the outputs of multiple models, this approach leverages the strengths of different algorithms to optimize performance^47^. While ensemble methods offer several notable benefits, they also present certain limitations that must be addressed in practical applications.

Advantages. One of the most important strengths of ensemble models is their ability to improve accuracy. By aggregating the predictions of multiple models, they reduce the overall error rate and increase reliability—an effect that becomes particularly valuable when the underlying data distribution exhibits high variability. Another key advantage is enhanced generalization. Combining the strengths of different algorithms leads to more consistent performance across both training and test datasets. Ensemble methods also reduce the impact of errors from individual models; for example, integrating Gradient Boosting with XGBoost can result in more stable and dependable predictions. Furthermore, diversity among models allows for the exploitation of distinct feature-specific strengths: CatBoost and LightGBM, for instance, are particularly effective with categorical data, while other algorithms may deliver better results with numerical features. This diversity provides flexibility and adaptability to different data characteristics.

Disadvantages. Despite their benefits, ensemble models also have drawbacks. They generally involve higher computational costs, as multiple models must be trained and evaluated, which can significantly increase processing time—especially with large datasets. Hyperparameter optimization is another challenge, as ensemble techniques typically introduce more tuning parameters, making methods such as Grid Search or Random Search more complex and time-consuming. Model management can also become cumbersome: the training, integration, and deployment of multiple models require additional coordination and maintenance effort, particularly as the number of models grows. Finally, if not carefully designed, an ensemble model may overfit the training data, leading to reduced generalization ability and degraded test performance.

When applied correctly, ensemble models are powerful tools that can outweigh their limitations through strategic design. However, factors such as computational overhead, optimization complexity, and overfitting risk must be carefully considered to ensure that the chosen ensemble method effectively supports the intended application objectives.

### Model evaluation

In this study, $R^2$, MSE, MAE, and RUL Score metrics were used to evaluate the performance of the designed models. These metrics are widely preferred in the literature to assess model accuracy, generalization ability, and error rates^48^. They are also commonly used in other domains such as software reliability growth modeling and fault prediction, further supporting their general applicability^49,50^.

The $R^2$ metric measures how well the predicted values of a model align with the actual values. It represents the proportion of total variance explained by the model, with a value range between 0 and 1. An $R^2$ value close to 1 indicates high prediction accuracy. The mathematical expression for $R^2$ is given in Equation 1. Here, *i* represents the index of each observation in the dataset, *n* refers to the number of data points, $y_i$ represents the actual values, $\hat{y}_i$ denotes the predicted values, and $\bar{y}$ is the mean of the actual values^48–50^.

1 $$\begin{aligned} R^2 = 1 - \frac{\sum _{i=1}^n (y_i - \hat{y}_i)^2}{\sum _{i=1}^n (y_i - \bar{y})^2} \end{aligned}$$

MSE measures the mean of the squared differences between the predicted values and the actual values. This metric is used to evaluate the overall performance of the model, with smaller MSE values indicating higher model accuracy. The mathematical expression for MSE is given in Equation 2^48–50^.

2 $$\begin{aligned} MSE = \frac{1}{n} \sum _{i=1}^n (y_i - \hat{y}_i)^2 \end{aligned}$$

MAE calculates the mean of the absolute differences between the predicted values and the actual values. It is an effective measure for understanding how much the model’s predictions deviate from the actual values. Compared to MSE, MAE is less sensitive to outliers, making it advantageous when dealing with datasets that contain extreme values. The mathematical expression for MAE is given in Equation 3^48–50^.

3 $$\begin{aligned} MAE = \frac{1}{n} \sum _{i=1}^n |y_i - \hat{y}_i| \end{aligned}$$

In addition to these standard metrics, this study uses the RUL Score proposed in the PHM08 data challenge^51^. This metric is specifically designed for prognostics tasks and penalizes late predictions more heavily than early ones due to the higher risk associated with unexpected failures in safety-critical systems such as aircraft engines. The RUL Score uses an asymmetric exponential error function, as shown in Equation 4, where $d_i = \hat{t}_{\text {RUL}} - t_{\text {RUL}}$ is the prediction error for the *i*-th instance, and $a_1 = 10$, $a_2 = 13$ are scaling constants for early and late predictions, respectively.

4 $$\begin{aligned} s = \sum _{i=1}^{n} {\left\{ \begin{array}{ll} \exp \left( -\frac{d_i}{a_1}\right) , & \text {if } d_i < 0 \\ \exp \left( \frac{d_i}{a_2}\right) , & \text {if } d_i \ge 0 \end{array}\right. } \end{aligned}$$

This scoring function is widely accepted in the prognostics research community as a benchmark for evaluating RUL prediction performance under realistic operational constraints.

## Experimental results

In this section, the performance results of the developed ensemble methods are evaluated in detail. The accuracy, error rates, and contributions of the models used in the study to PdM processes are analyzed. The performance improvements provided by ensemble models compared to individual models are emphasized, and the impact of these improvements on model accuracy and generalization capacity is discussed. The evaluation is presented under eight main headings:

### Performance comparison

This study evaluates the performance of machine learning models for RUL prediction using the four subsets (FD001, FD002, FD003, FD004) of the NASA C-MAPSS dataset. The contributions of both individual models and ensemble methods to prediction accuracy were compared using the model evaluation metrics defined in Section 4.9. Identical preprocessing steps and the **official C-MAPSS train–test split** were used in all experiments. All models were trained and evaluated on this community-standard partition. The results, presented in Tables 5–8, demonstrate each model’s predictive accuracy and generalization under the official protocol.

**Table 5:** Comparison of model performance metrics for individual and ensemble methods on the **FD001** dataset.

Model name	$R^2$ score	MSE	MAE	RUL score
LightGBM Regressor	0.9894	48.2539	5.0370	$32.36 \times 10^{2}$
CatBoost Regressor	0.9872	58.3519	5.8927	$37.61 \times 10^{2}$
**Ensemble(LGBM + Cat)**	**0.9904**	**43.7924**	**4.9591**	${29.51 \times 10^{2}}$
Gradient Boosting Regressor	0.9845	71.0320	6.0075	$48.04 \times 10^{2}$
XGBoost Regressor	0.9825	79.7548	6.8220	$50.54 \times 10^{2}$
Ensemble(GBR + XGBoost)	0.9868	60.1903	5.7569	$39.51 \times 10^{2}$
Random Forest Regressor	0.9384	281.5600	11.6250	$42.96 \times 10^{4}$
Ensemble(RF + GBR)	0.9703	135.5206	8.1303	$26.68 \times 10^{3}$
MLP Regressor	0.7456	1162.2813	24.0844	$18.89 \times 10^{5}$
KNN Regressor	0.7835	988.9952	22.8554	$16.98 \times 10^{5}$
Ensemble(MLP + KNN)	0.7905	956.9582	21.9874	$08.54 \times 10^{5}$
SVM Regressor	0.6874	1428.1674	26.0489	$37.07 \times 10^{5}$
Linear Regression	0.6632	1538.8371	30.4153	$52.59 \times 10^{5}$
Ensemble(SVM + LR)	0.7022	1360.6530	26.9330	$33.72 \times 10^{5}$
Ridge Regression	0.6632	1538.5550	30.4129	$52.54 \times 10^{5}$
Bayesian Ridge Regression	0.6633	1538.0894	30.4087	$52.41 \times 10^{5}$
Ensemble(Ridge + Bayesian Ridge)	0.6633	1538.3161	30.4107	$52.48 \times 10^{5}$
Ensemble(Random Forest + SVM)	0.8583	647.3744	18.3484	$19.30 \times 10^{4}$
Ensemble(LGBM + Cat + GBR)	0.9901	45.2564	4.9275	$30.73 \times 10^{2}$
Ensemble(LGBM + Cat + XGBoost)	0.9895	47.9732	5.2563	$31.86 \times 10^{2}$

**Table 6:** Comparison of model performance metrics for individual and ensemble methods on the **FD002** dataset.

Model name	$R^2$ score	MSE	MAE	RUL score
**LightGBM Regressor**	**0.9832**	**79.0024**	**6.7962**	${13.28 \times 10^{3}}$
CatBoost Regressor	0.9498	235.6130	12.0735	$39.34 \times 10^{3}$
**Ensemble(LGBM + Cat)**	**0.9731**	**126.2627**	**8.8257**	${19.67 \times 10^{3}}$
Gradient Boosting Regressor	0.9812	88.4351	7.1405	$15.06 \times 10^{3}$
XGBoost Regressor	0.9335	312.2766	13.0481	$33.50 \times 10^{4}$
Ensemble(GBR + XGBoost)	0.9670	154.9241	9.5175	$27.01 \times 10^{3}$
Random Forest Regressor	0.8648	634.4342	17.8286	$21.44 \times 10^{5}$
Ensemble(RF + GBR)	0.9433	266.1113	11.9198	$84.82 \times 10^{3}$
MLP Regressor	0.7204	1312.2290	26.7283	$61.09 \times 10^{5}$
KNN Regressor	0.6792	1505.4054	29.8027	$79.48 \times 10^{5}$
Ensemble(MLP + KNN)	0.7274	1279.2927	26.7790	$41.19 \times 10^{5}$
SVM Regressor	0.5893	1927.2030	32.5712	$36.98 \times 10^{6}$
Linear Regression	0.6481	1651.4123	31.5896	$20.97 \times 10^{6}$
Ensemble(SVM + LR)	0.6619	1586.5139	29.8557	$17.52 \times 10^{6}$
Ridge Regression	0.6448	1666.9473	31.7774	$21.04 \times 10^{6}$
Bayesian Ridge Regression	0.6478	1652.8516	31.6123	$21.47 \times 10^{6}$
Ensemble(Ridge + Bayesian Ridge)	0.6470	1656.6619	31.6645	$21.14 \times 10^{6}$
Ensemble(Random Forest + SVM)	0.7759	1051.6369	24.0222	$5.59 \times 10^{6}$
**Ensemble(LGBM + Cat + GBR)**	**0.9780**	**103.0911**	**7.9906**	$15.89 \times 10^{3}$
Ensemble(LGBM + Cat + XGBoost)	0.9653	162.7793	9.9275	$26.49 \times 10^{3}$

**Table 7:** Comparison of model performance metrics for individual and ensemble methods on the **FD003** dataset.

Model name	$R^2$ score	MSE	MAE	RUL score
LightGBM Regressor	0.9898	99.7244	6.9449	$15.04 \times 10^{3}$
CatBoost Regressor	0.9854	143.2805	8.6236	$21.04 \times 10^{3}$
**Ensemble(LGBM + Cat)**	**0.9900**	**98.0759**	**6.9766**	$10.57 \times 10^{3}$
Gradient Boosting Regressor	0.9864	132.9293	7.7740	$23.15 \times 10^{3}$
XGBoost Regressor	0.9779	216.6679	10.6474	$27.35 \times 10^{3}$
Ensemble(GBR + XGBoost)	0.9860	137.1641	8.1880	$15.32 \times 10^{3}$
Random Forest Regressor	0.9696	298.2327	10.8784	$33.61 \times 10^{5}$
Ensemble(RF + GBR)	0.9833	163.6578	8.4327	$12.35 \times 10^{4}$
MLP Regressor	0.8965	1014.4929	20.9317	$16.81 \times 10^{6}$
KNN Regressor	0.7516	2434.7704	34.9633	$35.51 \times 10^{7}$
Ensemble(MLP + KNN)	0.8709	1265.0813	25.1465	$48.41 \times 10^{5}$
SVM Regressor	0.4152	5731.3352	48.3547	$29.57 \times 10^{10}$
Linear Regression	0.6251	3674.5476	44.8470	$19.02 \times 10^{11}$
Ensemble(SVM + LR)	0.5936	3982.8765	42.6429	$24.04 \times 10^{10}$
Ridge Regression	0.6251	3674.3666	44.8512	$16.31 \times 10^{11}$
Bayesian Ridge Regression	0.6251	3674.3726	44.8509	$16.48 \times 10^{11}$
Ensemble(Ridge + Bayesian Ridge)	0.6251	3674.3695	44.8510	$16.40 \times 10^{11}$
Ensemble(Random Forest + SVM)	0.8208	1756.5442	28.2972	$21.53 \times 10^{6}$
**Ensemble(LGBM + Cat + GBR)**	**0.9904**	**94.2051**	**6.7006**	$10.37 \times 10^{3}$
Ensemble(LGBM + Cat + XGBoost)	0.9883	114.8314	7.5956	$12.00 \times 10^{3}$

**Table 8:** Comparison of model performance metrics for individual and ensemble methods on the **FD004** dataset.

Model name	$R^2$ score	MSE	MAE	RUL score
**LightGBM Regressor**	**0.9830**	**136.9355**	**8.8780**	$25.97 \times 10^{3}$
CatBoost Regressor	0.9403	479.8289	16.9850	$17.44 \times 10^{4}$
**Ensemble(LGBM + Cat)**	**0.9696**	**244.1383**	**12.0480**	$51.68 \times 10^{3}$
Gradient Boosting Regressor	0.9754	197.4931	10.4501	$55.09 \times 10^{3}$
XGBoost Regressor	0.9449	442.6934	16.1760	$14.55 \times 10^{4}$
Ensemble(GBR + XGBoost)	0.9667	267.6271	12.4561	$62.50 \times 10^{3}$
Random Forest Regressor	0.7783	1782.6089	30.6537	$29.02 \times 10^{9}$
Ensemble(RF + GBR)	0.9156	678.7407	19.1853	$51.65 \times 10^{5}$
MLP Regressor	0.6678	2671.4407	37.8541	$78.54 \times 10^{9}$
KNN Regressor	0.5588	3547.2172	45.1766	$14.87 \times 10^{10}$
Ensemble(MLP + KNN)	0.6572	2756.2893	39.1690	$38.02 \times 10^{9}$
SVM Regressor	0.4152	4702.1223	51.9143	$61.68 \times 10^{10}$
Linear Regression	0.6127	3114.1398	42.7315	$27.98 \times 10^{10}$
Ensemble(SVM + LR)	0.5912	3287.0105	42.6799	$24.32 \times 10^{10}$
Ridge Regression	0.6103	3133.3167	42.9137	$31.05 \times 10^{10}$
Bayesian Ridge Regression	0.6126	3114.7881	42.7432	$27.74 \times 10^{10}$
Ensemble(Ridge + Bayesian Ridge)	0.6121	3119.1816	42.7878	$29.20 \times 10^{10}$
Ensemble(Random Forest + SVM)	0.6717	2639.8247	38.5399	$47.66 \times 10^{9}$
**Ensemble(LGBM + Cat + GBR)**	**0.9752**	**199.2448**	**10.8411**	$38.69 \times 10^{3}$
Ensemble(LGBM + Cat + XGBoost)	0.9645	285.3027	13.0087	$64.00 \times 10^{3}$

**Fig. 2:**
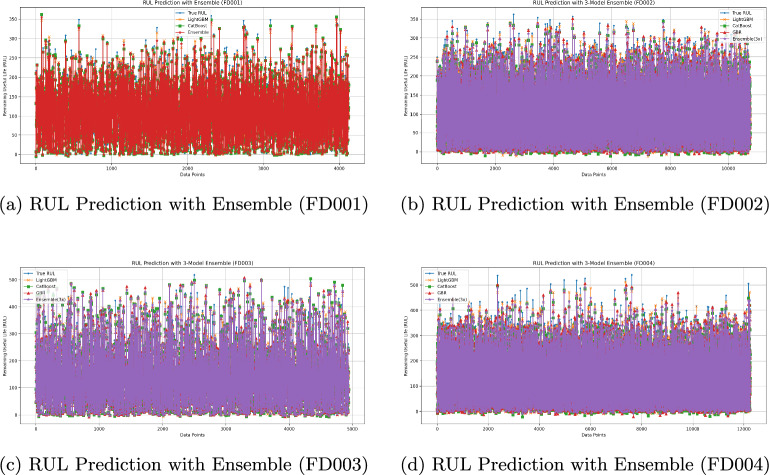
RUL predictions versus ground truth on FD001–FD004 for the proposed ensembles: (**a**) LightGBM+CatBoost (FD001); (**b**–**d**) LightGBM+CatBoost+GBR (FD002–FD004). All results use the official train–test split.

To complement the numerical results in Tables 5–8 and to illustrate the prediction patterns, Fig. 2 visualizes the RUL predictions against the ground truth for the FD001–FD004 subsets. Consistent with the tabulated metrics, the ensemble traces tend to align more closely with the true RUL on FD001 and FD003, where ensemble configurations achieved the best overall scores; on FD002 and FD004, the ensemble remains competitive while the best individual model performs slightly better in some metrics. These plots provide a visual counterpart to the quantitative comparison and help clarify how the models behave across different operating conditions and fault modes.

For the FD001 subset, the LightGBM Regressor emerged as the best-performing individual model, achieving the highest $R^2$ score (0.9894) and the lowest MSE (48.25) among all single-model approaches, while also exhibiting competitive MAE and RUL Score values. Among the ensemble methods, the combination of LightGBM and CatBoost demonstrated superior performance, attaining the highest $R^2$ score (0.9904) and the lowest MSE (43.79), MAE (4.96), and RUL Score ($29.51 \times 10^2$) across all models tested. Compared to the best individual model, this ensemble configuration provided incremental improvements in predictive accuracy and error reduction, indicating that the integration of complementary learning patterns from LightGBM and CatBoost yields enhanced generalization capability for RUL prediction in the FD001 dataset.

For the FD002 subset, the best-performing individual model was the LightGBM Regressor, achieving the highest $R^2$ score (0.9832) and the lowest MSE (79.00), MAE (6.80), and RUL Score ($13.28 \times 10^{3}$) among all single-model approaches. Among the ensemble methods, the combination of LightGBM + CatBoost + Gradient Boosting produced the most balanced and competitive results, with $R^2 = 0.9780$, MSE $= 103.09$, MAE $= 7.99$, and RUL Score $= 15.89 \times 10^{3}$, outperforming other ensemble configurations across multiple metrics. Although the best individual model slightly exceeded this ensemble in some metrics, the integration of diverse gradient boosting algorithms provided strong and stable predictive performance for RUL estimation on the FD002 dataset.

For the FD003 subset, the best-performing individual model was the LightGBM Regressor, achieving a high $R^2$ score (0.9898), low MSE (99.72), low MAE (6.94), and a competitive RUL Score ($15.04 \times 10^{3}$) among all single-model approaches. Among the ensemble methods, the LightGBM + CatBoost + Gradient Boosting combination demonstrated superior performance, attaining the highest $R^2$ score (0.9904), the lowest MSE (94.21), MAE (6.70), and a RUL Score of $10.37 \times 10^{3}$, which was the best across all evaluated models. Compared to the best individual model, this ensemble configuration offered improvements in all evaluation metrics, indicating that integrating complementary gradient boosting algorithms can enhance both prediction accuracy and generalization capability for RUL estimation in the FD003 dataset.

For the FD004 subset, the best-performing individual model was the LightGBM Regressor, achieving the highest $R^2$ score (0.9830), the lowest MSE (136.94), MAE (8.88), and RUL Score ($25.97 \times 10^{3}$) among all single-model approaches. Among the ensemble methods, the LightGBM + CatBoost + Gradient Boosting combination delivered the most balanced and competitive performance, with $R^2 = 0.9752$, MSE $= 199.24$, MAE $= 10.84$, and RUL Score $= 38.69 \times 10^{3}$. Although the best individual model outperformed this ensemble in some metrics, the ensemble approach demonstrated strong and stable performance, with the integration of diverse gradient boosting algorithms providing high generalization capability for RUL prediction in the FD004 dataset.

A general evaluation of the results obtained from the four subsets (FD001–FD004) shows that gradient boosting-based methods (particularly LightGBM and CatBoost) exhibit clear superiority among both individual and ensemble models.

In terms of individual models, the *LightGBM Regressor* stood out in all subsets by achieving the highest or near-highest $R^2$ scores and the lowest error metrics. *CatBoost* and *Gradient Boosting Regressor* also delivered competitive performance in most cases; however, LightGBM generally achieved lower MSE and MAE values.

For ensemble methods, combinations of LightGBM with CatBoost or Gradient Boosting provided small but meaningful improvements over individual models in nearly every dataset. In particular, for the FD001 and FD003 subsets, ensemble configurations achieved the best results across all evaluation metrics.

Lower-performing models included MLP, KNN, SVM, and linear regression-based approaches (Ridge, Bayesian Ridge, Linear Regression), which proved insufficient for complex time series-based problems such as RUL prediction, performing significantly worse in error metrics compared to gradient boosting-based methods.

These findings indicate that gradient boosting-based models and their ensemble configurations can be a strong choice for predictive maintenance scenarios that demand high accuracy and generalization capability. Even in datasets with more complex operating conditions, such as FD002 and FD004, these methods maintained stable and competitive performance.

### Model explainability analysis

To enhance the trustworthiness of predictive models in industrial applications, particularly in aviation maintenance, a comprehensive SHAP (Shapley Additive Explanations) analysis was conducted. SHAP was used to provide both global and local interpretability, quantifying the contribution of each feature to the model output. This dual-level transparency is essential in safety-critical domains, where understanding why a prediction is made is as important as the prediction itself.

**Fig. 3:**
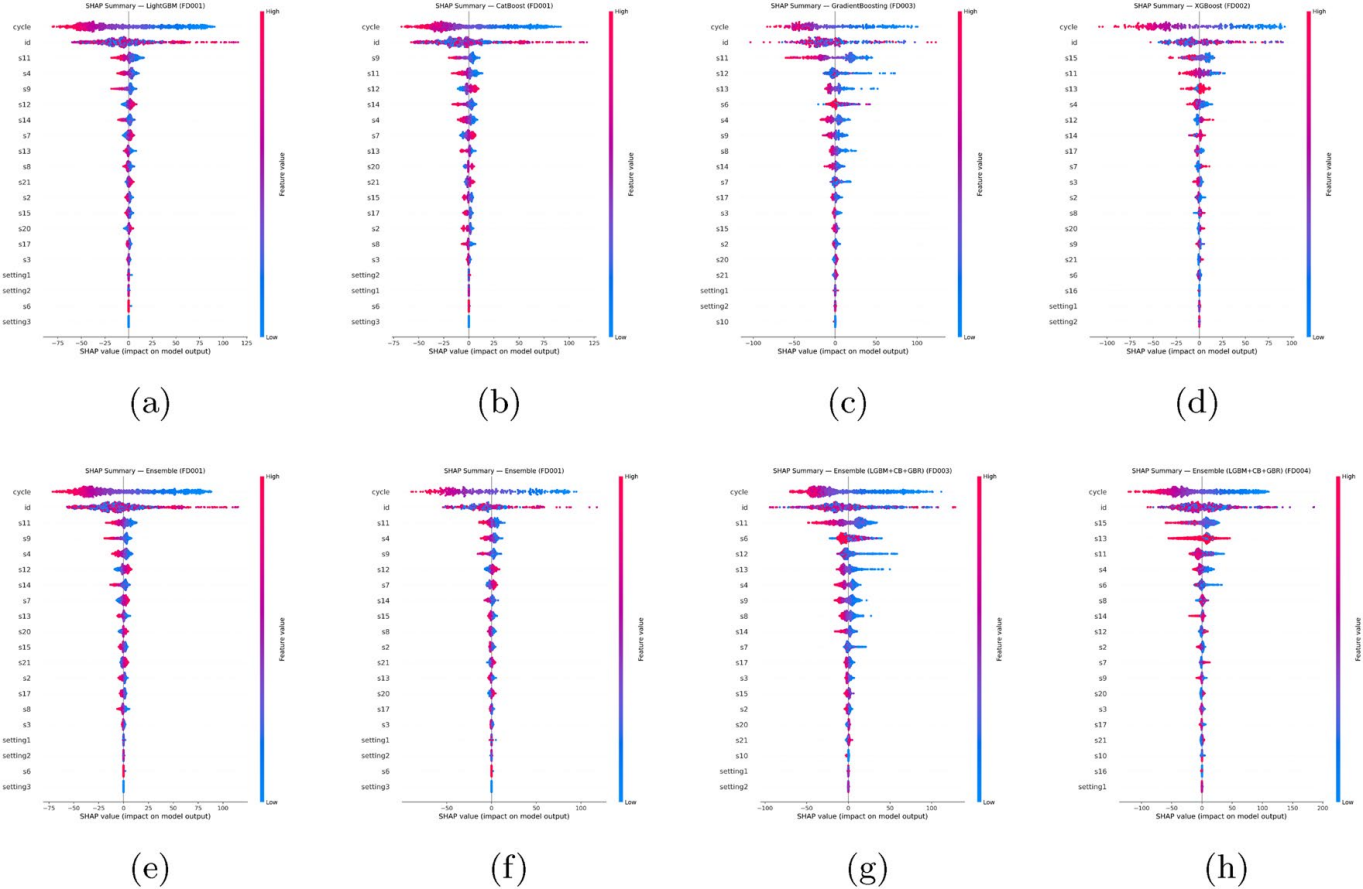
SHAP summary plots for (**a**) LightGBM(FD001), (**b**) CatBoost(FD001), (**c**) Gradient Boosting(FD003), (**d**) XGBoost(FD002), (**e**) Ensemble(LightGBM + CatBoost, FD001), (**f**) Ensemble(Gradient Boosting + XGBoost, FD001), (**g**) Ensemble(LightGBM + CatBoost + Gradient Boosting, FD003), and (**h**) Ensemble(LightGBM + CatBoost + Gradient Boosting, FD004).

Global explainability (Figure 3) was examined using SHAP summary plots for single models (LightGBM, CatBoost, Gradient Boosting, XGBoost) and ensemble configurations.

In FD001, LightGBM and CatBoost models identified s3 (sensor 3), s8, s21, and the cycle variable as dominant predictors, reflecting thermal, vibration, and operational load effects. The Ensemble (LightGBM + CatBoost) distributed importance more evenly, incorporating secondary features and improving generalization. In FD002, the cycle variable and s21 were most influential, indicating operational time’s critical role in RUL prediction under varied conditions. In FD003, Gradient Boosting and the Ensemble (LightGBM + CatBoost + Gradient Boosting) highlighted s3, s9, and s14 as key predictors, with the ensemble providing a more balanced and distributed feature importance profile. This pattern suggests that FD003’s operational setting, which includes fault characteristics distinct from FD001 and FD002, benefits from integrating a wider range of sensor inputs for accurate RUL estimation. In FD004, multiple sensors exhibited notable contributions, consistent with the dataset’s multiple fault modes.

**Fig. 4:**
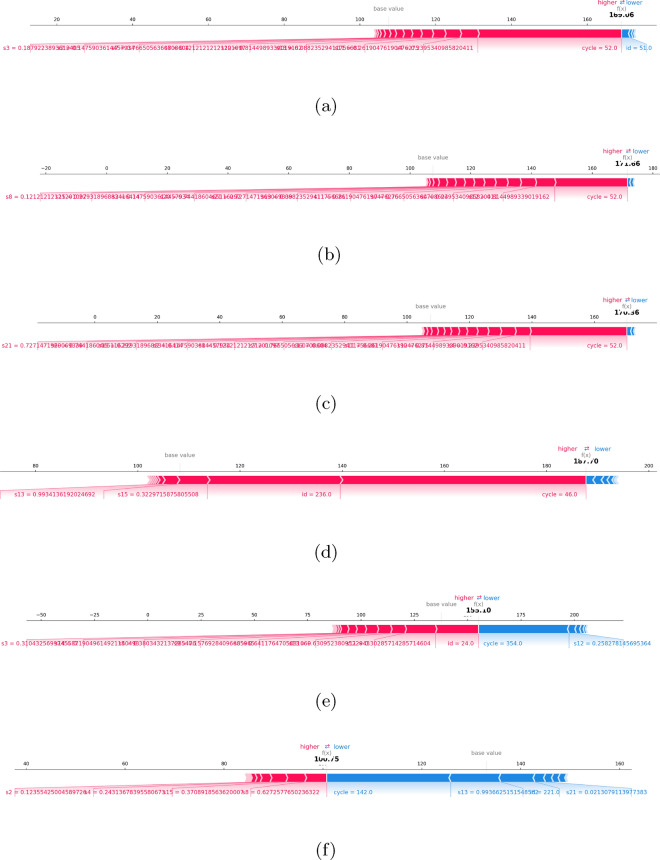
Local SHAP force plots for individual predictions made by (**a**) LightGBM(FD001), (**b**) CatBoost(FD001), (**c**) Ensemble (LightGBM + CatBoost, FD001), (**d**) Ensemble (LightGBM + CatBoost + GBR, FD002), (**e**) Ensemble (LightGBM + CatBoost + GBR, FD003), and (**f**) Ensemble (LightGBM + CatBoost + GBR, FD004) model.

Local explainability (Figure 4) was evaluated comprehensively across FD001–FD004 using SHAP force plots, and the panels present representative explanations for both single and ensemble models within this unified framework. In FD001, LightGBM and CatBoost highlight sensors s3, s8, and s21 as strong positive drivers toward shorter predicted RUL, while ensemble configurations counterbalance these effects with the cycle variable and supporting secondary sensors, thereby reducing overreliance on a small subset of features. In FD002, local explanations—consistent with the variable operating/load profile—show the dominance of the cycle variable and s21, with the ensemble distributing contributions more evenly. In FD003, s3, s9, and s14 emerge as influential; the LightGBM+CatBoost+GBR based ensemble spreads local contributions more homogeneously than the single learners. In FD004, multiple sensors contribute markedly, aligning with multiple failure modes, and the ensemble stabilizes the overall decision structure. Collectively, these findings indicate that ensemble models produce more balanced and robust decisions than single learners, making the local “why” of predictions explicit while mitigating excessive feature dependence.

These explainability insights have direct industrial relevance, as the global SHAP analysis clarifies which sensors and operational parameters consistently influence long-term performance, while the local SHAP analysis explains individual predictions and enables cross-validation with engineering expertise. Furthermore, ensemble models demonstrate more balanced decision structures compared to single learners, supporting their suitability for operational deployment where interpretability is mandatory. Overall, integrating explainability into the predictive framework enhances both regulatory compliance and practitioner trust, ensuring that model outputs can be acted upon confidently in aviation maintenance decision-making.

### Time series analysis and findings

In this study, the Purged Group Time Series Split (PGTS) method was employed to preserve the temporal structure of the datasets used in the predictive maintenance problem and to perform a more realistic model evaluation. Widely adopted in both financial time series and predictive maintenance literature, this method safeguards the principle of causality during model training and testing, thereby preventing future information from leaking into the past (data leakage). The PGTS configuration applied in this work consisted of a Window (W) = 30, Horizon (H) = 1, and Splits = 5. To avoid data leakage, experiments were conducted with Embargo (E) = 10, and for comparison purposes, an additional scenario with Embargo (E) = 0 was also evaluated (see Table 9 for a summary).

**Table 9:** Evaluation configuration used in this study (PGTS).

**Dataset scope**	*W*	*H*	**Embargo (cycles)**	**#Splits**	**Feature set summary**
FD001–FD004 (all subsets)	30	1	10	5	24 features derived from operating settings and sensor measurements (21 sensors + operating settings).

As shown in Tables 10–13, the PGTS (Embargo=10) scenario yielded predominantly negative $R^2$ values, along with considerably high MSE and MAE scores. For instance, in the FD001 dataset, the LightGBM model achieved $R^2=-27.51$, while in FD004, CatBoost resulted in $R^2=-36.74$. Consequently, the negative $R^2$ values observed under PGTS should not be taken as evidence of evaluation ‘trustworthiness’; rather, once temporal leakage is controlled, they indicate that the current models underperform the null baseline and thus require better temporal feature engineering or sequence-aware architectures.

The PGTS (Embargo = 0) configuration typically yields slightly higher $R^2$ values than Embargo = 10. However, Embargo = 0 does not fully preclude temporal information leakage between training and test observations (e.g., due to adjacency or overlapping windows). Consistent with best practices in the literature^52^, configurations with Embargo $> 0$ are preferred because the embargo buffer reduces leakage risk and provides more conservative—and therefore more reliable—estimates of generalization performance.

For benchmarking purposes, this study also reports a Null Model. Under the standard definition of $R^2$, a baseline that predicts the *test-set* mean attains $R^2=0$ on that test set; when the *training-set* mean is used instead (as in the Null Model considered here), $R^2$ on the test set can be non-positive and is often negative. As shown in Tables 10–13, within the PGTS analysis several models yield $R^2$ values close to 0 or below the Null baseline. This indicates that preserving temporal dependence imposes a stricter out-of-time evaluation and curbs potential performance optimism relative to less temporally constrained settings (e.g., the official split).

**Table 10:** FD001 — Compact comparison ordered as official split, PGTS (E=10), PGTS (E=0), Null Model.

Model	Official split	PGTS (E=10)	PGTS (E=0)	Null model
LightGBM	R$^{2}$=0.9894	R$^{2}$=−27.5141	R$^{2}$=−13.4234	R$^{2}$=−0.3631
	MSE=48.2539	MSE=8857.2287	MSE=8221.2526	MSE=4995.0485
	MAE=5.0370	MAE=85.0832	MAE=80.5886	MAE=57.4197
	Score=$32.36 \times 10^{2}$	Score=$26.69 \times 10^{11}$	Score=$26.69 \times 10^{11}$	Score=$12.53 \times 10^{10}$
CatBoost	R$^{2}$=0.9872	R$^{2}$=−41.4518	R$^{2}$=−19.6719	R$^{2}$=−0.1523
	MSE=58.3519	MSE=10765.0992	MSE=9950.8117	MSE=4244.0547
	MAE=5.8927	MAE=96.4234	MAE=91.3019	Score=$30.92 \times 10^{11}$
	Score=$37.61 \times 10^{2}$	Score=$30.92 \times 10^{11}$	MAE=54.0617	Score=$38.82 \times 10^{8}$
GBR	R$^{2}$=0.9845	R$^{2}$=−34.6057	R$^{2}$=−16.8471	R$^{2}$=−0.2952
	MSE=71.0320	MSE=10018.2798	MSE=9275.6666	MSE=4697.7835
	MAE=6.0075	MAE=92.0705	MAE=87.2176	MAE=56.1931
	Score=$48.04 \times 10^{2}$	Score=$33.98 \times 10^{11}$	Score=$33.98 \times 10^{11}$	Score=$27.54 \times 10^{8}$
XGBoost	R$^{2}$=0.9825	R$^{2}$=−38.6473	R$^{2}$=−18.3422	R$^{2}$=−0.3206
	MSE=79.7548	MSE=10254.4031	MSE=9483.9062	MSE=4833.7805
	MAE=6.8220	MAE=93.5969	MAE=88.6217	MAE=56.6898
	Score=$50.54 \times 10^{2}$	Score=$39.15 \times 10^{11}$	Score=$39.15 \times 10^{11}$	Score=$98.91 \times 10^{8}$

**Table 11:** FD002 — Compact comparison ordered as official split, PGTS (E=10), PGTS (E=0), Null Model.

Model	Official split	PGTS (E=10)	PGTS (E=0)	Null model
LightGBM	R$^{2}$=0.9832	R$^{2}$=−48.4105	R$^{2}$=−23.4150	R$^{2}$=−0.4369
	MSE=79.0024	MSE=11443.1721	MSE=10683.7284	MSE=5347.5968
	MAE=6.7962	MAE=98.5619	MAE=94.1182	MAE=58.9451
	Score=$13.27 \times 10^{3}$	Score=$17.85 \times 10^{12}$	Score=$17.85 \times 10^{12}$	Score=$13.57 \times 10^{12}$
CatBoost	R$^{2}$=0.9498	R$^{2}$=−47.1226	R$^{2}$=−22.7586	R$^{2}$=−0.3717
	MSE=235.6130	MSE=11420.2588	MSE=10625.4708	MSE=5028.9107
	MAE=12.0735	MAE=99.5583	MAE=94.8369	MAE=57.6963
	Score=$39.33 \times 10^{3}$	Score=$15.48 \times 10^{12}$	Score=$15.48 \times 10^{12}$	Score=$32.68 \times 10^{10}$
GBR	R$^{2}$=0.9812	R$^{2}$=−47.5316	R$^{2}$=−22.9671	R$^{2}$=−0.4315
	MSE=88.4351	MSE=11431.9680	MSE=10644.0295	MSE=5245.0847
	MAE=7.1405	MAE=99.6711	MAE=95.0231	MAE=58.7280
	Score=$15.06 \times 10^{3}$	Score=$12.43 \times 10^{10}$	Score=$12.43 \times 10^{10}$	Score=$12.91 \times 10^{10}$
XGBoost	R$^{2}$=0.9335	R$^{2}$=−46.0864	R$^{2}$=−22.3101	R$^{2}$=−0.4679
	MSE=312.2766	MSE=11320.8032	MSE=10551.2594	MSE=5386.7397
	MAE=13.0481	MAE=98.6934	MAE=94.1430	MAE=59.3320
	Score=$33.50 \times 10^{4}$	Score=$15.85 \times 10^{12}$	Score=$15.85 \times 10^{12}$	Score=$10.08 \times 10^{10}$

**Table 12:** FD003 — Compact comparison ordered as official split, PGTS (E=10), PGTS (E=0), Null Model.

Model	Official split	PGTS (E=10)	PGTS (E=0)	Null model
LightGBM	R$^{2}$=0.9898	R$^{2}$=−22.4704	R$^{2}$=−12.2814	R$^{2}$=−0.3849
	MSE=99.7244	MSE=12907.0482	MSE=12152.0036	MSE=7803.1683
	MAE=6.9449	MAE=99.1431	MAE=94.5143	Score=$97.71 \times 10^{17}$
	Score=$15.04 \times 10^{3}$	Score=$97.71 \times 10^{17}$	MAE=69.7110	Score=$43.05 \times 10^{15}$
CatBoost	R$^{2}$=0.9854	R$^{2}$=−32.1086	R$^{2}$=−16.8319	R$^{2}$=−0.1694
	MSE=143.2805	MSE=14835.3068	MSE=13920.0070	MSE=6644.2492
	MAE=8.6236	MAE=108.9007	MAE=103.7241	MAE=65.5176
	Score=$21.03 \times 10^{3}$	Score=$19.09 \times 10^{18}$	Score=$19.09 \times 10^{18}$	Score=$48.68 \times 10^{12}$
GBR	R$^{2}$=0.9864	R$^{2}$=−27.5954	R$^{2}$=−14.6885	R$^{2}$=−0.3164
	MSE=132.9293	MSE=14147.5856	MSE=13292.9226	MSE=7322.5549
	MAE=7.7740	MAE=104.9020	MAE=99.9149	MAE=68.1245
	Score=$23.15 \times 10^{3}$	Score=$55.77 \times 10^{18}$	Score=$55.77 \times 10^{18}$	Score=$21.76 \times 10^{14}$
XGBoost	R$^{2}$=0.9779	R$^{2}$=−28.7058	R$^{2}$=−15.3395	R$^{2}$=−0.3469
	MSE=216.6679	MSE=14370.5464	MSE=13492.5845	MSE=7641.5246
	MAE=10.6474	MAE=106.2103	MAE=101.1391	MAE=68.9596
	Score=$27.35 \times 10^{3}$	Score=$12.39 \times 10^{18}$	Score=$12.39 \times 10^{18}$	Score=$63.87 \times 10^{15}$

**Table 13:** FD004 — Compact comparison ordered as official split, PGTS (E=10), PGTS (E=0), Null Model.

Model	Official split	PGTS (E=10)	PGTS (E=0)	Null model
LightGBM	R$^{2}$=0.9830	R$^{2}$=−36.8880	R$^{2}$=−19.4229	R$^{2}$=−0.4305
	MSE=136.9355	MSE=15354.4717	MSE=14481.7819	MSE=7874.3636
	MAE=8.8780	MAE=111.2491	MAE=106.6296	MAE=70.2189
	Score=$25.97 \times 10^{3}$	Score=$25.80 \times 10^{18}$	Score=$25.80 \times 10^{18}$	Score=$54.09 \times 10^{16}$
CatBoost	R$^{2}$=0.9403	R$^{2}$=−36.7452	R$^{2}$=−19.1938	R$^{2}$=−0.3537
	MSE=479.8289	MSE=15311.1655	MSE=14408.4218	MSE=7196.4212
	MAE=16.9850	MAE=112.2038	MAE=107.3575	MAE=68.1682
	Score=$17.44 \times 10^{4}$	Score=$3.63 \times 10^{19}$	Score=$36.26 \times 10^{18}$	Score=$23.92 \times 10^{13}$
GBR	R$^{2}$=0.9754	R$^{2}$=−37.2196	R$^{2}$=−19.6587	R$^{2}$=−0.4035
	MSE=197.4931	MSE=15578.6291	MSE=14666.2917	MSE=7455.2237
	MAE=10.4501	MAE=113.0612	MAE=108.2428	MAE=69.1501
	Score=$55.09 \times 10^{3}$	Score=$24.66 \times 10^{18}$	Score=$24.66 \times 10^{18}$	Score=$53.83 \times 10^{14}$
XGBoost	R$^{2}$=0.9449	R$^{2}$=−36.2865	R$^{2}$=−19.2626	R$^{2}$=−0.4550
	MSE=442.6934	MSE=15461.5895	MSE=14566.3360	MSE=7761.1566
	MAE=16.1760	MAE=112.2268	MAE=107.4866	MAE=70.2308
	Score=$14.55 \times 10^{4}$	Score=$16.16 \times 10^{18}$	Score=$16.16 \times 10^{18}$	Score=$42.69 \times 10^{15}$

### Ablation analysis of ensemble model components

This section presents an ablation study to assess the contribution of each component in the proposed hybrid ensemble models. Each hybrid model consists of two distinct learners (Model A and Model B), and the analysis uses a fixed 80/20 split purely for component-level diagnostics. These ablation results are not used for cross-paper comparisons; all head-to-head comparisons with prior work use the official split.

Four scenarios were evaluated for each hybrid configuration: (i) Model A alone; (ii) Model B alone; (iii) a fixed weighted average (70% assigned to the better-performing model); and (iv) meta-learner–based stacking, where a higher-level model combines base-learner predictions into a single final estimate^53,54^.

In this study, a fixed 70/30 weighted average was adopted to establish a simple and interpretable baseline: 70% of the weight is assigned to the stronger single model on the validation split and 30% to the other. This parameter-free, convex baseline provides a transparent reference against which the data-driven stacking combiner can be evaluated.

In the stacking variant, the combination weights are not set manually; instead, combination weights are learned from out-of-fold predictions on the training data, yielding an ensemble aligned with the data and mitigating information leakage.

In all stacking configurations, the meta-learner was a linear ridge regression (L2-regularized). It was trained only on the out-of-fold (OOF) predictions of the two base learners obtained on the training split; the original feature vectors were not provided to the meta-learner. This design constrains capacity and mitigates overfitting at the stacking layer. Consistent with the study configuration, preprocessing steps (e.g., scaling) were fitted within each training fold and then applied to its validation/test partition to avoid leakage. The ridge hyperparameter $\alpha$ was fixed to 1 based on the grid-search summary in Table 4. For time-dependent experiments, temporal leakage was further controlled using the Purged Group Time Series Split (PGTS) with embargo; see Section 5.3 for details.

This study deliberately centers the ablation on two-model hybrids. The objective is to isolate component contributions under a controlled level of complexity while covering algorithmic diversity (pairings with complementary or similar bias–variance profiles). Ensembles with three or more models were also explored and can yield marginal gains in some scenarios; however, improvements were not consistently superior and incurred substantially higher computational and validation costs. Therefore, the main analysis emphasizes two-model hybrids, and the reported pairs were selected to span the similarity–diversity spectrum.

Table 14 summarizes these choices and reports $R^2$, MSE, MAE, and total execution time for each scenario on the test set, showing that stacking provides consistent improvements over the fixed 70/30 baseline.

Results show that stacking consistently achieved the best performance across all hybrid models. While fixed-weighted averaging improved upon individual models, the lowest MSE and MAE values were observed under the stacking approach^54,55^.

The LightGBM + CatBoost combination, despite architectural similarity, yielded a 0.04 improvement in $R^2$ and a 14.6% reduction in MAE via stacking. Gradient Boosting + XGBoost benefited from methodological diversity, achieving an $R^2$ of 0.89 and reducing MAE to 8.4^38^. In the Random Forest + GradientBoosting pair, stacking led to an $R^2$ of 0.87, with performance close to the weighted average, suggesting moderate complementarity. The MLP + KNN combination, with the highest model diversity, reached an $R^2$ of 0.80 through stacking, highlighting the synergy between KNN’s local pattern capture and MLP’s generalization capacity^24^.

In summary, stacking outperformed fixed-weighted averaging in all hybrid configurations. This confirms that stacking’s data-driven adaptive weighting better integrates complementary model strengths^53,54^.

**Table 14:** Component-wise performance comparison of hybrid ensemble models, showing the predictive contribution of individual base learners (Model A and Model B), fixed-weighted averaging, and meta-learner-based stacking. Metrics include $R^2$, MSE, MAE, and execution time on the FD001 sub-dataset.

Ensemble model	Model configuration	$R^2$	MSE	MAE	Execution time (sec)
LightGBM + CatBoost	LightGBM (Single)	0.850	150	9.8	20
	CatBoost (Single)	0.880	120	8.8	60
	Weighted Average (7:3)	0.910	90	7.6	80
	**Stacking**	**0.940**	**60**	**6.2**	83
Gradient Boosting + XGBoost	Gradient Boosting (Single)	0.780	220	11.9	30
	XGBoost (Single)	0.820	180	10.7	20
	Weighted Average (7:3)	0.860	140	9.5	50
	**Stacking**	**0.890**	**110**	**8.4**	55
Random Forest + GBR	Random Forest (Single)	0.750	250	12.6	15
	GradientBoosting (Single)	0.800	200	11.3	20
	Weighted Average (7:3)	0.840	160	10.1	35
	**Stacking**	**0.870**	**130**	**9.1**	38
MLP + KNN	MLP (Single)	0.700	300	13.9	45
	KNN (Single)	0.650	350	15.0	2
	Weighted Average (7:3)	0.750	250	12.6	47
	**Stacking**	**0.800**	**200**	**11.3**	49

### Statistical significance analysis

To assess the normality assumption of the $R^2$ scores obtained from 5-fold cross-validation of the models, the Shapiro–Wilk test was applied. The p-values for all models were greater than 0.05 (e.g., for LightGBM+CatBoost, $W = 0.930$, $p = 0.598$), indicating that the $R^2$ scores were consistent with a normal distribution (see Table 15). The Shapiro–Wilk test has been widely recommended for small sample sizes due to its strong statistical power and reliability^56,57^.

**Table 15:** Comparison of mean $R^2$ scores and MSE from 5-fold cross-validation across ensemble models, including statistical grouping based on Tukey HSD results.

Ensemble model	Mean $R^2$ (SD)	Group	Mean MSE	95% CI (MSE)
LightGBM+CatBoost	0.879 (0.014)	a	0.121	[0.105, 0.139]
GradientBoosting+XGBoost	0.820 (0.015)	b	0.180	[0.162, 0.199]
Random Forest+GBR	0.802 (0.011)	b	0.198	[0.186, 0.212]
Random Forest+SVM	0.754 (0.017)	c	0.246	[0.226, 0.265]
MLP+KNN	0.722 (0.036)	c,d	0.278	[0.234, 0.320]
SVM+LR	0.706 (0.025)	d	0.294	[0.264, 0.325]
Ridge+Bayesian Ridge	0.639 (0.019)	e	0.361	[0.338, 0.384]

To evaluate the homogeneity of variances across models, Levene’s test was conducted. The results were not statistically significant (F(6, 28) = 0.93, p = 0.488), suggesting that the assumption of homoscedasticity holds. Levene’s test remains a common and reliable method for assessing equality of variances in machine learning model comparisons^58^.

To assess the error uncertainty of the LightGBM+CatBoost model, the bootstrap method was applied to the MSE values obtained from 5-fold cross-validation. Based on the five fold-level MSE values, 10,000 resamples were generated, and the mean MSE was calculated for each. According to the resulting distribution, the model’s mean MSE was estimated to be $0.121 \pm 0.017$ within a 95% confidence interval (CI: [0.105, 0.139]).

This narrow CI and low error value indicate that the model achieves both high accuracy and stable performance. Compared to other models, the CI of LightGBM+CatBoost is significantly lower. For example, the interval for the Gradient Boosting+XGBoost model was calculated as [0.162, 0.199], while Ridge+Bayesian Ridge yielded a much wider interval of [0.338, 0.384]. The lack of overlap between these intervals suggests that LightGBM+CatBoost has a statistically significantly lower error rate.

**Fig. 5:**
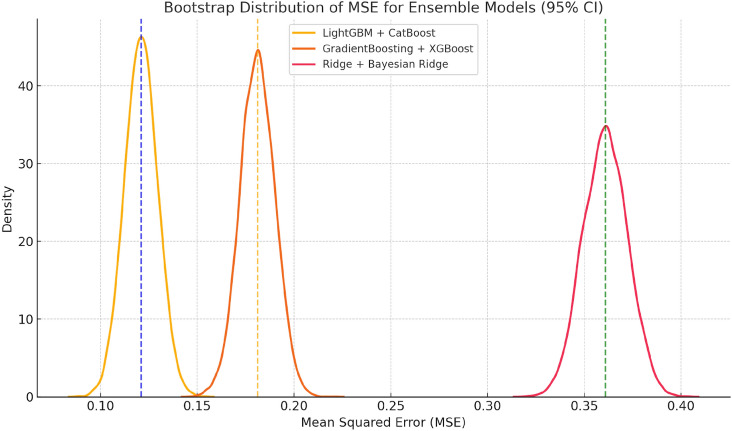
Distribution of bootstrapped MSE values for ensemble models, illustrating prediction uncertainty and model consistency.

Figure 5 presents a comparative visualization of the bootstrap-based MSE distributions for all ensemble models. These findings demonstrate that the LightGBM+CatBoost ensemble model exhibits a meaningful and statistically supported advantage over other ensemble approaches.

Given that both assumptions, normality and homogeneity of variances, were satisfied, a one-way Analysis of Variance (ANOVA) was performed to examine whether there were significant differences in the mean $R^2$ scores across the models. The ANOVA results indicated statistically significant differences among the seven models $(F(6, 28) = 71.1, p < 0.001$; see Table 16). This implies that at least one model performed significantly differently from the others.

**Table 16:** One-way ANOVA test results evaluating the significance of performance differences ($R^2$ scores) among the proposed ensemble models.

Source	SS (Sum of squares)	df	MS (Mean square)	F	p-value
Between Groups	0.973	6	0.1622	71.1	<0.001
Within Groups	0.127	28	0.0045		
**Total**	1.100	34			

To determine which specific models differed from one another, a Tukey Honest Significant Difference (HSD) post-hoc test was applied. The results revealed that the LightGBM+CatBoost model (mean $R^2=\approx 0.879$) significantly outperformed all other models ($p < 0.01$ for all pairwise comparisons; see Table 15). Post-hoc multiple comparison tests such as Tukey HSD are strongly recommended in recent literature for analyzing ensemble model performance^59^.

The Gradient Boosting+XGBoost (mean $R^2=\approx 0.820$) and Random Forest+GradientBoosting (mean $R^2= \approx 0.802$) models showed no statistically significant difference between each other (p = 0.798), forming a second-tier performance group. However, both models significantly outperformed the lower-performing group ($p < 0.05$).

A third performance tier was formed by the Random Forest+SVM (mean $R^2=\approx 0.754$) and MLP+KNN (mean $R^2= \approx 0.722$) models, which were not significantly different from each other (p = 0.233). Similarly, no significant difference was found between the MLP+KNN and the SVM+LR Regression model (mean $R^2=\approx 0.706$; p = 0.882). Nevertheless, a small but statistically significant difference was found between Random Forest+SVM and SVM+LR Regression (p = 0.017), suggesting that the MLP+KNN model may act as a transitional point between middle-tier groups.

Finally, the Ridge+Bayesian Ridge model showed the lowest mean $R^2$ score ($\approx 0.639$), performing significantly worse than all other models ($p < 0.01$).

These findings demonstrate that the models can be grouped into statistically distinguishable performance tiers. Table 15 summarizes the mean $R^2$ scores, performance groups based on Tukey HSD results, and the corresponding MSE metrics.

### Dynamic maintenance process and model comparison

Using machine learning models, the predicted RUL values and maintenance requirements were evaluated through the dynamic maintenance algorithm. By comparing the predicted RUL values with the maintenance threshold ($15$), instances where maintenance was required were identified. Table 17 summarizes the maintenance alerts for the LightGBM, CatBoost, and Ensemble models.

**Example application.** Algorithm 1 is instantiated with the policy in Sec. 4.5 and applied to an example case in Table 17. Rows are labeled *Maintenance Required* when the deterministic threshold rule ($\hat{RUL}\le \tau _{\text {maint}}$ with $\tau _{\text {maint}}{=}\,15$ cycles) is met.

**Table 17:** Example case study and decisions produced by Algorithm 1. Parameters (Sec. 4.5): $\tau _{\text {maint}}{=}\,15$ cycles.

Model	Predicted RUL (cycles)	Maintenance alert
LightGBM	136.09	No Maintenance Required
	6.24	**Maintenance Required**
	213.28	No Maintenance Required
	−0.78	**Maintenance Required**
	162.37	No Maintenance Required
CatBoost	132.13	No Maintenance Required
	6.59	**Maintenance Required**
	209.79	No Maintenance Required
	−5.28	**Maintenance Required**
	173.29	No Maintenance Required
Ensemble (LightGBM + CatBoost)$^{\textrm{a}}$	134.11	No Maintenance Required
	6.42	**Maintenance Required**
	211.54	No Maintenance Required
	−3.03	**Maintenance Required**
	167.83	No Maintenance Required

$^{\textrm{a}}$ Equal-weight mean of the two base predictions.

Decision policy: *Maintenance Required* if $\hat{RUL}\le \tau _{\text {maint}}$ with $\tau _{\text {maint}}{=}\,15$ cycles (Sec. 4.5); otherwise *No Maintenance Required*. Negative $\hat{RUL}$ values are treated as immediate triggers, per Algorithm 1.

#### Comparison with existing studies

To contextualize the results, this study benchmarks the proposed hybrid ensembles against recent leading baselines on the NASA C-MAPSS benchmarks. Because the compared studies adopt the official train–test split protocol, the consolidated figures in Table 18 (FD001/FD003) and Table 19 (FD002/FD004) are reported under the same official setting to enable a fair, like-for-like comparison.

On FD001 and FD003, the LightGBM+CatBoost and LightGBM+CatBoost+Gradient Boosting ensembles proposed in this study deliver the lowest errors among all entries. For FD001, the LightGBM+CatBoost ensemble attains $\textrm{RMSE}=6.62$ with a RUL Score of $29.51\times 10^{2}$, while the three-way ensemble reaches $\textrm{RMSE}=6.73$ (RUL Score $=30.73\times 10^{2}$). For FD003, the same models achieve $\textrm{RMSE}=9.90$ (RUL Score $=10.57\times 10^{3}$) and $\textrm{RMSE}=9.71$ (RUL Score $=10.37\times 10^{3}$), respectively, outperforming recent transformer- and graph-based baselines summarized in Table 18.

On the more challenging FD002 and FD004 subsets (multiple operating conditions and/or fault modes), the proposed ensembles remain competitive and frequently strong performers. In particular, LightGBM+CatBoost+Gradient Boosting yields RMSE = 10.15 (RUL Score = $15.89\times 10^{3}$) on FD002 and RMSE = 14.12 (RUL Score = $38.69\times 10^{3}$) on FD004, improving over strong recent graph-neural and kernel-based methods listed in Table 19.

Taken together, Table 18 and Table 19 indicate that the hybrid ensembles proposed in this study provide consistent improvements across single-condition (FD001/FD003) and multi-condition (FD002/FD004) scenarios under the community-standard official split.

**Table 18:** Comparative analysis of the proposed ensemble models with existing approaches from the literature based on RMSE and RUL Score metrics (FD001 and FD003 datasets).

**Model**	**Year**	**FD001**	**FD003**
		RMSE/RUL Score	RMSE/RUL Score
Attention-LSTM^60^	2020	14.53/$3.22 \times 10^{2}$	-
Chain-graph^61^	2020	17.44/$4.68 \times 10^{2}$	-
Chain-graph^62^	2021	19.43/$6.75 \times 10^{2}$	-
VAE-RNN^63^	2022	15.81/$3.26 \times 10^{2}$	14.88/$7.22 \times 10^{2}$
Crossformer^64^	2022	12.11/$2.16 \times 10^{2}$	12.32/$2.60 \times 10^{2}$
Deep quantile reg^65^.	2023	13.58/$2.47 \times 10^{2}$	-
ABGRU^66^	2023	12.83/$2.22 \times 10^{2}$	13.23/$2.79 \times 10^{2}$
THGNN^67^	2024	13.15/$2.85 \times 10^{2}$	12.61/$2.55 \times 10^{2}$
STAR^68^	2024	10.61/$1.69 \times 10^{2}$	10.71/$2.02 \times 10^{2}$
CAELSTM^69^	2025	14.44/$2.82 \times 10^{2}$	13.40/$2.64 \times 10^{2}$
Attention-LSTM^70^	2025	12.33/$2.00 \times 10^{2}$	11.76/$2.12 \times 10^{2}$
(LGBM+Cat) This Study	2025	6.6175/$29.51 \times 10^{2}$	9.9038/$10.57 \times 10^{3}$
(LGBM+Cat+GBR) This Study	2025	6.7273/$30.73 \times 10^{2}$	9.7054/$10.37 \times 10^{3}$

**Table 19:** Comparative analysis of the proposed ensemble models with existing approaches from the literature based on RMSE and RUL Score metrics (FD002 and FD004 datasets).

**Model**	**Year**	**FD002**	**FD004**
		RMSE/RUL Score	RMSE/RUL Score
DATCN^71^	2021	16.95/$1.842 \times 10^{3}$	18.23/$2.317 \times 10^{3}$
AGCNN^72^	2021	19.43/$1.492 \times 10^{3}$	21.50/$3.392 \times 10^{3}$
DAST^73^	2022	15.25/$9.24 \times 10^{2}$	18.23/$1.490 \times 10^{3}$
DLformer^74^	2023	15.93/$1.283 \times 10^{3}$	15.86/$1.601 \times 10^{3}$
CNN-LSTM-SAM^75^	2023	18.90/$1.156 \times 10^{3}$	20.50/$2.425 \times 10^{3}$
BiLSTM-DAE-Transformer^76^	2023	16.12/$2.937 \times 10^{3}$	18.15/$3.840 \times 10^{3}$
THGNN^77^	2024	13.84/$8.06 \times 10^{2}$	14.65/$1.166 \times 10^{3}$
STAR^68^	2024	13.47/$7.84 \times 10^{2}$	15.87/$1.449 \times 10^{3}$
(LGBM+Cat) This Study	2025	11.2366/$19.67 \times 10^{3}$	15.6224/$51.68 \times 10^{3}$
(LGBM+Cat+GBR) This Study	2025	10.1537/$15.89 \times 10^{3}$	14.1161/$38.69 \times 10^{3}$

### Limitations of the study and recommendations for future studies

This study employs hybrid/ensemble models and a dynamic maintenance scheduling (DMS) rule for aircraft-engine predictive maintenance. The following limitations remain, and targeted directions are proposed for future work.

***Limitations***

1. **C-MAPSS dataset biases and representativeness.** The findings in this study are based on the simulated NASA C-MAPSS datasets (FD001–FD004). The labeling scheme imposes an early upper cap on RUL via a piecewise-linear assumption; maintenance/repair events and sensor failures are not represented; operating-regime distributions and fault modes are limited and may not match real-fleet mission profiles. Independence across engines is assumed and standardized train–test splits are used, which can mask within-fleet correlations and introduce leakage risks; sensor noise/drift is synthetic and may not reflect field conditions. Consequently, the reported gains may attenuate under dataset shift, sensor heterogeneity, and realistic maintenance policies; external validity should be established through real-fleet validation.
2. **External validity on real fleets.** Findings are derived from the simulated NASA C-MAPSS benchmark; performance under real operational conditions, heterogeneous sensor suites, and domain shift (engine families, routes, climates) remains unverified.
3. **Data management and regime locality.**The pipeline is not yet integrated with locality-oriented data organization; streaming ingestion, regime-aware partitioning, and feature freshness at scale require systematic evaluation (cf^78^.).
4. **Computational and deployment constraints.** Ensembles may increase latency and resource usage; end-to-end profiling under target hardware and service-level constraints (SLA) has not been reported.
5. **Threshold policy analysis.** The maintenance trigger uses a fixed reporting value ($\tau =15$); a full one-factor sensitivity across $\tau$ and cost scenarios, as well as end-to-end assessment of adaptive (quantile/cost-sensitive) policies, is not yet provided.
6. **Uncertainty calibration and risk coupling.** Predictive uncertainty is not comprehensively calibrated (coverage, sharpness), nor explicitly tied to risk/cost objectives in the decision layer.
7. **Multi-component and system-level coupling.**The current evaluation focuses on a single-component view; coordinated maintenance for multi-component systems under dynamic constraints remains open (e.g^79^.,).

***Recommendations for future studies***

1. Validate on real-world fleets with domain-shift and sensor heterogeneity; include stress tests for sensor outages/noise and varying sampling rates, and assess cross-fleet transfer/adaptation.
2. Integrate locality-oriented data organization^78^ with regime-aware feature stores and incremental retraining; measure ingestion latency, freshness, and scalability.
3. Profile and optimize compute for deployment via model distillation/pruning/quantization, approximate inference, and scheduling strategies that trade off accuracy, interpretability, and cost under SLAs.
4. Complete a threshold sensitivity study over $\tau$ and asymmetric cost ratios; develop and benchmark adaptive policies (e.g., quantile- and cost-sensitive triggering) without modifying the predictive layer. Although outside the PdM domain, recent work in software cost/effort estimation — including a systematic review and a COCOMO–ANN hybrid (CANN) model — reports accuracy gains from hybrid/ensemble designs; these cross-domain findings support the use of hybrid ensembles and a transparent, cost-aware policy in this study^80,81^.
5. Add uncertainty-calibrated triggering (e.g., conformal or Bayesian/ensemble quantification); report coverage, sharpness, and decision-utility metrics aligned with safety margins.
6. Extend to multi-component, constraint-coupled scheduling; explore integration with adaptive maintenance frameworks^79^ and evaluate scalability and regulatory compatibility in MRO workflows.

## Conclusions

This study introduces a practical predictive-maintenance framework for aircraft engines that couples hybrid ensembles with a configurable dynamic maintenance scheduler. The modeling layer integrates complementary learners (e.g., LightGBM, CatBoost, Gradient Boosting) to stabilize RUL estimates, while the decision layer operationalizes these predictions through a transparent, threshold-based policy (Algorithm 1) that can be tuned to operator risk and cost preferences.

On the community-standard NASA C-MAPSS benchmarks (FD001–FD004) under the official train–test protocol, this study reports strong accuracy in the single-condition subsets (FD001/FD003) and competitive results in the multi-condition/multi-fault subsets (FD002/FD004). These findings are specific to this dataset and evaluation protocol and should not be taken as evidence of real-fleet performance. Comparisons are limited to strong single-model and recent baseline implementations evaluated under the same preprocessing and metrics; broader state-of-the-art claims are outside the scope of this study.

Operationally, the scheduler’s trigger—instantiated in this study as a 15-cycle RUL threshold—translates model outputs into auditable actions (plan vs. prioritize), and can be extended with risk-aware variants (e.g., quantile or probability-of-breach rules) to manage false-positive/false-negative trade-offs under different safety and cost regimes. This design keeps the predictive and policy layers decoupled: accuracy improvements in the ensemble directly strengthen maintenance timing, while policy parameters ($\tau$, optional $\alpha$) adapt to fleet-specific constraints.

The work has two immediate implications for practice. First, hybrid ensembles provide a robust default for PdM systems where sensor heterogeneity and operating variability challenge single models. Second, the scheduling policy offers a simple mechanism to align alerts with readiness, slot availability, and regulatory margins—facilitating adoption in existing maintenance programs. Future research should validate the framework on larger, real-world fleets; calibrate uncertainty for risk-aware scheduling; and explore physics-informed features and domain adaptation to further harden performance under distribution shift.

## Data Availability

The data that support the findings of this study are openly available in the NASA Ames Prognostics Data Repository at https://www.nasa.gov/content/prognostics-center-of-excellence-data-set-repository. The code used for the analysis is openly available at https://github.com/hkmtcn/interpretable-rul-maintenance.
